# Expanding the base editing scope to GA and relaxed NG PAM sites by improved xCas9 system

**DOI:** 10.1111/pbi.13259

**Published:** 2019-10-02

**Authors:** Chengwei Zhang, Wen Xu, Feipeng Wang, Guiting Kang, Shuang Yuan, Xinxin Lv, Lu Li, Ya Liu, Jinxiao Yang

**Affiliations:** ^1^ Beijing Key Laboratory of Maize DNA Fingerprinting and Molecular Breeding Beijing Academy of Agriculture & Forestry Sciences Beijing China

**Keywords:** base editing, cytosine base editor, xCas9, tRNA‐sgRNA, rice

Dear Editor,

Base editors have been developed to be powerful tools to generate precise point mutations. However, their applications are hindered by the strict canonical NGG PAM requirement of *Streptococcus pyogenes* Cas9 (SpCas9). Cas effectors recognizing different PAMs or relaxed PAMs have been employed to address this limitation. Recently, xCas9 and Cas9‐NG were both used to further broaden the editing scope to NG PAM sites (Hu *et al*., 2018; Nishimasu *et al*., 2018). xCas9 showed broader PAM recognition including GAA and GAT than Cas9‐NG and has been employed in base editors in human cells (Hu *et al*., 2018). However, no detected sites with these PAMs were edited by reported xCas9 involved cytosine base editors (xCas9‐CBEs) in rice (Hua *et al*., 2019; Li *et al*., 2019; Ren *et al*., 2019; Zhong *et al*., 2019). In this study, we generated an efficient xCas9‐CBE to achieve C‐to‐T mutation at GAA, GAT and even GAC, GAG PAM sites in rice, expanding the targeting scope and providing a reference for other plants and animals.

We first analyzed all reports on xCas9‐CBEs and found that tRNA–sgRNA system was never used. Since xCas9 is a high‐fidelity SpCas9 variant (Zhong *et al*., 2019), it may require perfectly matched 20‐nucleotide (nt) target like other high‐fidelity Cas9 variants (Zhang *et al*., 2017). Studies showed that tRNA–sgRNA system can produce precise 20‐nt sequence completely complementary to the targets by the cellular enzymes RNase P and RNase Z (Figure 1a). Considering tRNA–sgRNA system can also improve sgRNA expression level (Xie *et al*., 2015; Zhang *et al*., 2017), and the scaffold modified sgRNA (known as esgRNA) was reported to enhance editing efficiency (Chen *et al*., 2013), we chose tRNA–sgRNA system including two sgRNA forms (Figure 1a) to develop xCas9‐CBEs in rice.

**Figure 1:**
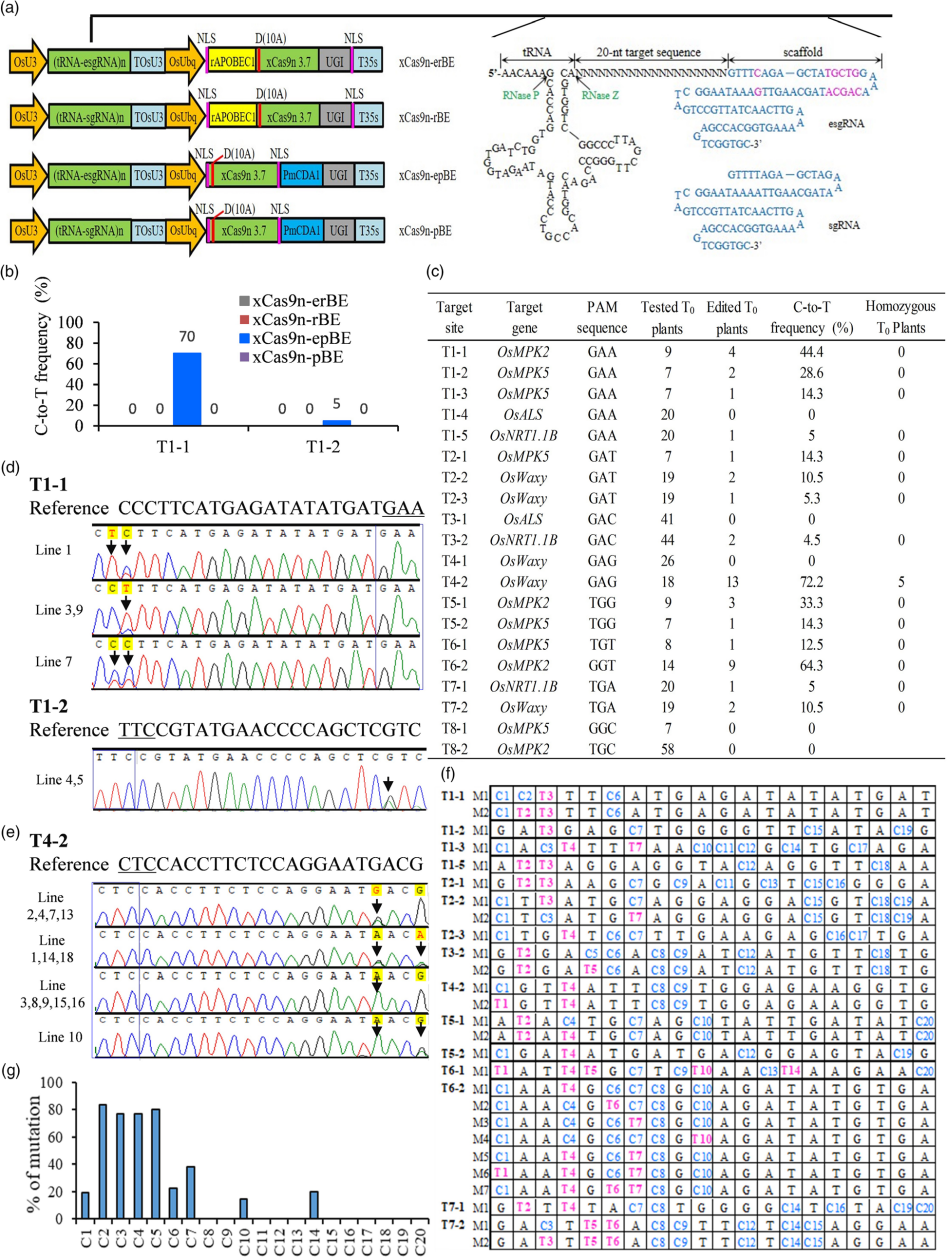
Cytosine base editing by xCas9n‐epBE in rice. (a) Schematic view of xCas9n‐erBE, xCas9n‐rBE, xCas9n‐epBE and xCas9n‐pBE base editors. erBE/rBE: rAPOBEC1 based cytosine base editor (CBE) with or without esgRNA. epBE/pBE: PmCDA1 based CBE with or without esgRNA. n in (tRNA‐esgRNA)n or (tRNA‐sgRNA)n means the target number in the vector. The sequences of tRNA‐esgRNA/sgRNA units are given. tRNA is cleaved by RNase P and RNase Z at specific sites. Different nucleotides between the scaffold of esgRNA and sgRNA are marked in pink. (b) Efficiency comparison of four base editors at two genomic sites, T1‐1 and T1‐2, in rice resistant calli. Number of detected calli is 20. (c) Base editing efficiencies of xCas9n‐epBE in rice T_0_ plants. Homozygous T_0_ plant was designated when all C‐to‐T mutations were homozygous. T1 to T8 represent the target sites with different PAMs. (d) Sequencing chromatograms at T1‐1 and T1‐2 sites of all mutated T_0_ lines produced by xCas9n‐epBE. Arrows indicate the edited bases. The PAM sequences are underlined or shown in blue boxes. G‐to‐A conversions in the opposite strand are shown for the edited lines at T1‐2 targets. (e) Sequencing chromatograms at T4‐2 sites of all mutated T_0_ lines produced by xCas9n‐epBE. G‐to‐A conversions in the opposite strand are shown. Lines 3, 8, 9, 15 and 16 are homozygous substitution plants. Arrows point to the edited bases. (f) Mutant genotypes of the edited lines at 15 target sites. M indicates on‐target mutant genotype. Ts shown in pink at these specific positions mean C‐to‐T conversions, counting from the 5’ end of the target. (g) Ratio of T_0_ plants with mutation at certain position to all the edited T_0_ plants containing C at such position. *X*‐axis represents the position of C from the 5’ end of the target.

A262T/R324L/S409I/E480K/E543D/M694I/E1219V mutations were introduced into rice codon‐optimized SpCas9 (Wu *et al*., 2019) to generate xCas9 3.7. We then fused the D10A nickase of xCas9 3.7 (xCas9n 3.7) with the commonly used rat cytidine deaminase rAPOBEC1 or *Petromyzon marinus* cytidine deaminase1 (PmCDA1) and with uracil DNA glycosylase inhibitor (UGI) and subsequently placed them under control of the *O. sativa* ubiquitin (OsUbq) promoter in tRNA‐sgRNA or tRNA‐esgRNA system, generating four xCas9‐CBEs designated as xCas9n‐erBE, xCas9n‐rBE, xCas9n‐epBE and xCas9n‐pBE, respectively (Figure 1a). We first chose two GAA PAM sites from two mitogen‐activated protein kinases (*OsMPK2* and *OsMPK5*) genes to test the systems. Rice resistant calli and stable transgenic T_0_ plants were generated by callus selection under hygromycin B after *Agrobacterium*‐mediated transformation of rice as previously described (Wu *et al*., 2019). The data from rice resistant calli showed only xCas9n‐epBE displayed base editing activity (Figure 1b). In T_0_ plants, xCas9n‐epBE also showed robust base editing activity, with mutation rates of 44.4% and 28.6%, respectively at these two sites (Figure 1c,d). These results suggest that xCas9n‐epBE might be a promising cytosine base editor for GAA and GAT PAM sites in rice.

To further confirm the efficacy of xCas9n‐epBE on targets with GAA and GAT PAMs, we tested the C‐to‐T substitution capability at another three GAA PAM target sites (T1‐3, T1‐4 and T1‐5) in the *OsMPK5*,* OsALS* and *OsNRT1.1B* genes, respectively and three GAT PAM sites (T2‐1, T2‐2 and T2‐3) in the *OsMPK5* or *OsWaxy* genes. In T_0_ plants, at three GAA PAM sites, both T1‐3 and T1‐5 were edited with frequencies of 14.3% and 5%, respectively, while T1‐4 was not edited (Figure 1c). Among all three target sites with GAT PAM, xCas9n‐epBE also showed detectable base mutations, with frequencies ranging from 5.3% to 14.3% (Figure 1c). Collectively, these findings demonstrate that xCas9n‐epBE can act as an effective cytosine base editor for genomic sites with GAA and GAT PAMs in rice.

Since the target sites harbouring GAA and GAT PAMs can be edited by xCas9n‐epBE, we hypothesized that xCas9n‐epBE could work at GAN PAM sites, including the other two PAMs, GAC and GAG. To test this, we used xCas9n‐epBE to edit two GAC PAM target sites (T3‐1 and T3‐2) and two GAG PAM target sites (T4‐1 and T4‐2) in the *OsALS*,* OsNRT1.1B* or *OsWaxy* genes (Figure 1c). Each had one site being edited in T_0_ plants (Figure 1c). xCas9n‐epBE showed robust editing activity at the GAG T4‐2 site (Figure 1e), with frequencies over 70%, and showed low mutation frequency of 4.5% at the GAC T3‐2 site (Figure 1c). Therefore, xCas9n‐epBE could further broaden the targeting scope of cytosine base editing from GAA and GAT PAM sites to GA PAM sites in rice.

The previously reported xCas9‐CBEs were not efficient at NG PAM sites in rice regenerated plants, with the exception of canonical NGG PAM sites (Hua *et al*., 2019; Li *et al*., 2019; Ren *et al*., 2019; Zhong *et al*., 2019). Given the good performance of xCas9n‐epBE at editing sites with GA PAM, we envisioned that xCas9n‐epBE could also work on NG PAM sites in rice. Two target sites for each PAM (NGG, NGT, NGA and NGC) were tested (Figure 1c). xCas9n‐epBE could edit all six targets with NGG, NGT and NGA PAMs, with frequencies ranging from 5% to 64.3% (Figure 1c), but for two NGC PAM sites, no mutation was detected from seven and 58 transgenic plants (Figure 1c), possibly because the sites were suboptimal. These results show that xCas9n‐epBE also enable efficient base editing at target sites with relaxed NG PAMs in rice, suggesting that the effective xCas9n‐epBE outperforms the previously reported xCas9‐CBEs and might act as a good alternative to Cas9‐NG involved CBE in rice. tRNA‐esgRNA system might play an important role and might significantly improve the editing frequency to make xCas9n‐epBE be effective.

Finally, for further analyzing the editing window, editing preference and mutation types of xCas9n‐epBE, all 44 edited T_0_ plants of the above 15 edited target sites were collected. Analyzed results showed that the editing window typically spanned positions 1 to 7 within the protospacer and expanded to 10 and 14 occasionally (Figure 1f). In the editing window, C2, C3, C4 and C5 have priority to be edited over C1, C6 and C7 normally (Figure 1g). C10 and C14 also displayed comparable performance to C1, C6 or C7 (Figure 1g). Furthermore, homozygous mutations were seldom generated (Figure 1c,e).

In summary, we have successfully developed an effective cytosine base editor, xCas9n‐epBE, using tRNA‐esgRNA system in rice. xCas9n‐epBE enabled efficient C‐to‐T conversion at sites containing broadened GA PAM or even relaxed NG PAM, which largely expanded the base editing target scope in rice. The editing window, editing preference and mutation types have also been clearly presented for effective application. Moreover, tRNA‐esgRNA expression system might function well on xCas9 based gene disruption and adenine base editing in rice.

## Acknowledgements

This work was supported by Beijing Academy of Agriculture & Forestry Sciences. The funding included Innovative Team Construction Project of BAAFS (JNKYT201603) and the Beijing Scholars Program (BSP041).

## Conflict of interest

The authors submitted patent applications based on the results reported in this paper.

## Author contributions

J.Y. and Y.L. designed the experiments. C.Z., W.X., F.W., G.K., S.Y., X.L. and L.L. performed all the experiments. C.Z. and W.X. analyzed the results. C.Z., W.X., Y.L. and J.Y. wrote the manuscript. J.Y. and Y.L. supervised the project.
